# Utilization of Surveillance after Polypectomy in the Medicare Population – A Cohort Study

**DOI:** 10.1371/journal.pone.0110937

**Published:** 2014-11-13

**Authors:** Iris Lansdorp-Vogelaar, Stacey Fedewa, Chun Chieh Lin, Katherine S. Virgo, Ahmedin Jemal

**Affiliations:** 1 Department of Public Health, Erasmus MC, Rotterdam, the Netherlands; 2 Surveillance & Health Services Research, Intramural Research Department, American Cancer Society, Atlanta, GA, United States of America; 3 Department of Epidemiology, Rollins School of Public Health, Emory University, Atlanta, GA, United States of America; 4 Department of Health Policy and Management, Emory University, Atlanta, GA, United States of America; University Hospital Llandough, United Kingdom

## Abstract

**Background:**

Surveillance in patients with previous polypectomy was underused in the Medicare population in 1994. This study investigates whether expansion of Medicare reimbursement for colonoscopy screening in high-risk individuals has reduced the inappropriate use of surveillance.

**Methods:**

We used Kaplan-Meier analysis to estimate time to surveillance and polyp recurrence rates for Medicare beneficiaries with a colonoscopy with polypectomy between 1998 and 2003 who were followed through 2008 for receipt of surveillance colonoscopy. Generalized Estimating Equations were used to estimate risk factors for: 1) failing to undergo surveillance and 2) polyp recurrence among these individuals. Analyses were stratified into three 2-year cohorts based on baseline colonoscopy date.

**Results:**

Medicare beneficiaries undergoing a colonoscopy with polypectomy in the 1998–1999 (n = 4,136), 2000–2001 (n = 3,538) and 2002–2003 (n = 4,655) cohorts had respective probabilities of 30%, 26% and 20% (p<0.001) of subsequent surveillance events within 3 years. At the same time, 58%, 52% and 45% (p<0.001) of beneficiaries received a surveillance event within 5 years. Polyp recurrence rates after 5 years were 36%, 30% and 26% (p<0.001) respectively. Older age (≥ 70 years), female gender, later cohort (2000–2001 & 2002–2003), and severe comorbidity were the most important risk factors for failure to undergo a surveillance event. Male gender and early cohort (1998–1999) were the most important risk factors for polyp recurrence.

**Conclusions:**

Expansion of Medicare reimbursement for colonoscopy screening in high-risk individuals has not reduced underutilization of surveillance in the Medicare population. It is important to take action now to improve this situation, because polyp recurrence is substantial in this population.

## Introduction

Individuals in whom adenomas have been detected are considered to be at increased risk of developing colorectal cancer (CRC), even after the adenomas have been removed [1]. These high-risk individuals are therefore recommended to undergo regular surveillance with colonoscopy (every five years if 1–2 adenomas smaller than 1 cm, every 3 years otherwise) [2]. Colonoscopic polypectomy and subsequent surveillance have been estimated to reduce CRC incidence by 76–90% [1] and mortality by 53% [3] in adenoma patients. In patients aged 50 years and older, surveillance after adenoma removal is the single most common indication for colonoscopy [4]. Two studies evaluating the utilization of surveillance colonoscopy in adenoma patients were recently published [5], [6]. Both studies concluded that surveillance colonoscopy was overused in low-risk subjects, while concurrently being underused in higher-risk subjects.

The subjects in the aforementioned studies showing underutilization were volunteers in the polyp prevention trial [5] and the Prostate, Lung, Colorectal and Ovarian Cancer screening trial [6]. Volunteers in clinical trials are known to be more health conscious and may consequently be more desirous and demanding of frequent colonoscopy examinations. Their colonoscopy utilization patterns may therefore not be representative of the general US population and may underestimate the true problem of underuse in the population, while at the same time overestimating overuse. SEER-Medicare is generally representative of the elderly (65+ year-old) population and will as such provide better insight into the under- and overuse of surveillance in the general population, at least those aged 65 years and older, representing 67% of CRC [7].

A previous study of surveillance patterns in the Medicare population showed that 25% of patients with previous polypectomy did not undergo a surveillance event within 5 years [8]. At the same time a high likelihood of polyp recurrence of more than 50% within 5 years was observed in those with surveillance, underscoring the need for compliance with surveillance recommendations. On the other hand, more than 50% of patients with previous polypectomy received surveillance within the shortest recommended surveillance interval of 3 years. This study was conducted in a cohort of Medicare beneficiaries in 1994, before coverage of colonoscopy screening for high-risk individuals including adenoma patients was introduced in 1998. In the current study, we investigated whether expansion of Medicare reimbursement for colonoscopy screening in high-risk individuals has affected the under- and overutilization of surveillance in this population.

## Methods

### Data Source

The study population consisted of the 5% sample cohort of Medicare beneficiaries without cancer who reside in SEER areas obtained along with the SEER-Medicare database, a collaborative effort of the National Cancer Institute, the SEER registries, and the Centers for Medicare & Medicaid Services [9], [10]. All non-cancer SEER-Medicare enrollees (aged 66 and older) with full coverage in Part A, Part B and no Health Maintenance Organization (HMO) coverage for 24 consecutive months after a claim for a colonoscopy with index polypectomy between 1998 and 2003 were included in the study and followed until December 31, 2008. To ensure that colonoscopies were being performed for surveillance purposes, we excluded individuals with a cancer diagnosis before or at baseline colonoscopy or two claims of any one of the following diagnoses (for which colonoscopy may constitute part of work up) indicated 12 months before polypectomy: colitis and enteritis (International Classification of Diseases, Ninth Revision, Clinical Modification (ICD-9) codes 009.0, 009.1, 555.1-555.2, 555.9, 556, 558), iron deficiency anemia (280.9), chronic vascular insufficiency of intestine (557.1), unspecified intestinal obstruction (560.9), diverticula (562.1), stenosis (569.2), hemorrhage (569.3, 578.9), ulceration (569.41, 569.82), colostomy and enterostomy complications (569.6), perforation (569.83), filling defects (793.4), rupture (537.83–537.89), or other disorders of the intestine (546.81–546.89, 569.85–569.86).

### Analysis

To assess under- and over-utilization of surveillance in individuals with polypectomy, we selected beneficiaries with at least a baseline polypectomy between 1998 and 2003. Polypectomy claims were identified by searching outpatient and physician/supplier claims files, using Health Care Common Procedure Coding System (HCPCS) codes 45383, 45384, 45385; and ICD-9 codes 45.42, 45.43, 48.36. We used Kaplan-Meier analysis to estimate the probability of undergoing a subsequent surveillance event over time. A surveillance event was defined as follow-up by colonoscopy (HCPCS codes G0105, G0121, 45378, 45380, 45383, 45384, 45385; and ICD-9 codes 45.23, 45.25, 45.42, 45.43, 48.36), sigmoidoscopy (HCPCS codes G0104, 45330, 45331, 45333, 45338, 45339, 45300–45320; and ICD-9 codes 45.24, 48.23, 48.24) or barium enema (HCPCS codes G0106, G0120, 74270, 74280). To investigate changes over time, for example because of expansion of reimbursement in 2001, the analysis was stratified by time of baseline colonoscopy: 1998–1999, 2000–2001 and 2002–2003. Individuals were included in a cohort if they had a polypectomy within the selected time period. The baseline colonoscopy was defined as the first colonoscopy with polypectomy for an individual within the selected time period. As such, one individual could contribute to all three cohorts, if this person had a colonoscopy with polypectomy in each of the specified time periods. However, an individual could only contribute once within a cohort. Time was measured from the baseline colonoscopy to the time of subsequent surveillance event or censoring because of:

1. End of enrollment of fee-for-service Part A or B, or enrollment in HMO
2. Two diagnoses of any of the above-mentioned comorbidities following index polypectomy
3. Death or
4. End of follow-up period (December 31, 2008)

To account for subjects brought back early for clinical concerns, repeat colonoscopy, sigmoidoscopy or barium enema examinations performed within 6 months of the baseline colonoscopy were considered part of the baseline procedure and potential polypectomies at those examinations were included with the baseline results. Appendix S1 provides an overview of how patients included in the study were followed over time and included in the analyses.

Kaplan-Meier survival curves were used to estimate utilization of surveillance in the Medicare population with previous colonoscopy with polypectomy over time. Survival curves were also used to estimate polyp recurrence rates for only those subjects who had a surveillance event combined with a polypectomy. We used 5-year survival estimates as the basis for comparison between cohorts. In addition, we used Generalized Estimating Equations (GEE) to estimate risk factors for failure to undergo surveillance within 5 years in this population. We also used GEE to estimate risk factors for subsequent polypectomy during surveillance in all patients that had surveillance, accounting for patient-level clustering as patients were allowed to contribute to more than one cohort [11]. We could not use logistic regression because observations were not completely independent due to repeated measures for the same patient. Patient characteristics considered in the model included age, race/ethnicity, gender, Charlson comorbidity score (including comorbidities developed after baseline colonoscopy) [12], and urban versus rural status.

All analyses were performed using SAS software (version 9.2; SAS Institute Inc, Cary, NC).

### Sensitivity Analysis

We performed the following sensitivity analyses:

1. Inclusion of patients with polyps detected and removed at (procto-) sigmoidoscopy (HCPCS: 45333, 45338, 45339, 45308, 45309, 45315, 45320)
2. Single inclusion of individuals in the cohort of their first colonoscopy with polypectomy between 1998 and 2003. (Individuals contributed to a single cohort)
3. Limiting the definition of a surveillance event to a colonoscopy and
4. Including patients diagnosed with CRC from the SEER-Medicare data between 1998-2003 who had at least 1 polypectomy more than 6 months before their diagnosis date.

### IRB approval

The Institutional Review Board of Morehouse School of Medicine determined the study appropriate for exemption under federal regulations.

## Results

There were 3,538 Medicare beneficiaries with a polypectomy in 1998–1999, 4,136 in 2000–2001 and 4,655 in 2002–2003 fulfilling our inclusion criteria. The characteristics of the population for each of these cohorts are presented in Table 1. Approximately 55% of the patients in each cohort were women and the vast majority lived in urban areas. The age and race distribution differed between cohorts, with the 1998–1999 cohort having relatively more white people and people between ages 66–69 years than the 2000–2001 and 2002–2003 cohorts. Approximately 65% of the population had or developed a Charlson comorbidity score of 1 or more. Of the people that received a surveillance event, around 98% received a colonoscopy.

**Table 1:** Characteristics of study population, N (%).

Characteristics	1998–1999 cohort N = 3,538	2000–2001 cohort N = 4,136	2002–2003 cohort N = 4,655	Total N = 10,852
Sex				
Female	1,955 (55.3)	2,353 (56.9)	2,505 (53.8)	6,104 (56.3)
Male	1,583 (44.7)	1,783 (43.1)	2,150 (46.2)	4,748 (43.8)
Age¥				
66–69	639 (18.1)	426 (10.3)	297 (6.4)	1,068 (9.8)
70–74	1,106 (31.3)	1,498 (36.2)	1,617 (34.7)	3,661 (33.7)
75–79	978 (27.6)	1,231 (29.8)	1,558 (33.5)	3,348 (30.9)
80–84	529 (15.0)	645 (15.6)	815 (17.5)	1,830 (16.9)
85+	286 (8.1)	336 (8.1)	368 (7.9)	945 (8.7)
Race¥				
White	3,003 (84.9)	3,443 (83.2)	3,830 (82.3)	9,022 (83.1)
Black	203 (5.7)	184 (4.5)	241 (5.2)	565 (5.2)
Other/Unknown*	332 (9.4)	509 (12.3)	584 (12.6)	1,265 (11.7)
Urban/Rural				
Urban	3,477 (98.3)	4,075 (98.5)	4,570 (98.2)	10,668 (98.3)
Rural/Missing*	61 (1.7)	61 (1.4)	85 (1.8)	184 (1.7)
Charlson comorbidity score†				
0	1,242 (35.1)	1,462 (35.4)	1,587 (34.1)	3,767 (34.7)
1	848 (24.0)	982 (23.7)	1,090 (23.4)	2,565 (23.6)
2 or more	1,448 (40.9)	1,692 (40.9)	1,978 (42.5)	4,520 (41.7)
If surveillance event, type of event				
Barium enema	37 (1.6)	27 (1.0)	29 (1.3)	84 (1.4)
Colonoscopy	2,269 (97.5)	2,385 (97.9)	2,167 (97.5)	5,862 (97.5)
Sigmoidoscopy	21 (0.9)	24 (1.0)	26 (1.2)	64 (1.1)

¥ Statistically significant difference (p<0.01) between 98–99, 00–01 and 02–03 cohort.

* There were 31 beneficiaries with unknown race, and 32 with missing urban/rural status.

† Including comorbidities developed within 5 years after baseline colonoscopy (or until censoring or event).

**Figure 1:**
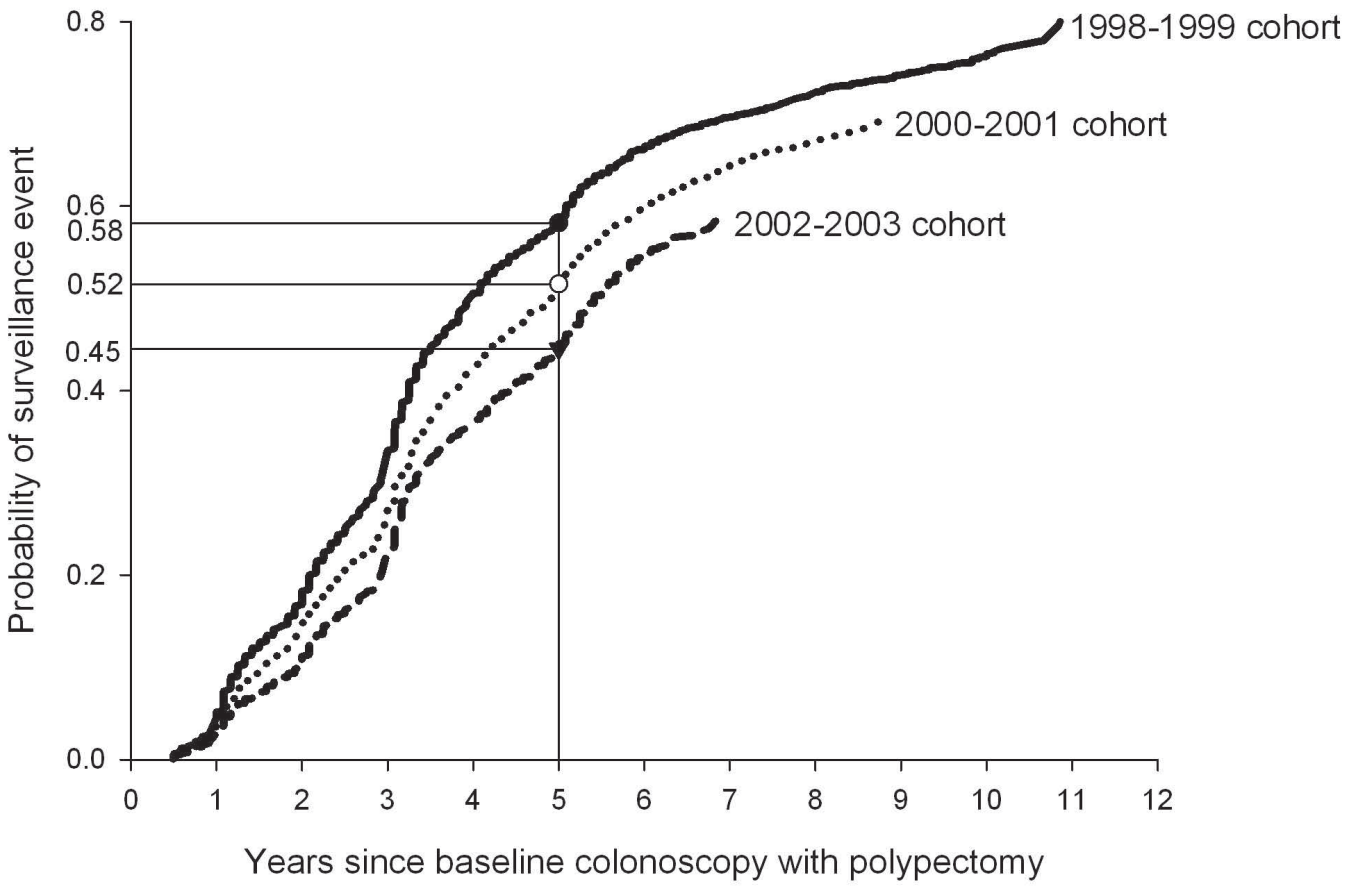
Kaplan-Meier estimates of probability of first surveillance event, stratified by cohort based on date of baseline colonoscopy with polypectomy.

Patients without a surveillance event were followed for a mean period of 5.1–6.4 years, depending on cohort. Among the 6,985 patients with surveillance, 47% of surveillance events occurred within 3 years, and 83% within 5 years. Mean follow-up until surveillance was 3.2–3.4 years. A Kaplan-Meier probability curve of surveillance utilization is presented in Figure 1. The cumulative probability of a surveillance event within three years decreased from 31.5% in the 1998–1999 cohort to 20.0% in the 2002–2003 cohort (p<0.001). At the same time, however, the cumulative probability of a subsequent surveillance event within 5 years also significantly decreased from 58% to 45% respectively (p<0.001). Consequently, the probability of failure to undergo a surveillance event within 5 years increased from 42% to 55% in this period.

GEE regression identified older age, female gender, later cohort and high comorbidity as factors associated with failure to undergo subsequent surveillance (Table 2). The highest odds ratios were found for older age (increasing to 3.7 [95% CI: 3.0–4.4] for people aged 85 years and older compared to people aged 66–69 years) and severe comorbidity (increasing to 2.2 [95% CI: 2.0–2.4] for a comorbidity score of 2+ compared to those without comorbidity) (p<0.001). Rural status and race were not significantly associated with failure to undergo subsequent colonoscopy.

**Table 2:** Odds ratio for failing to undergo a subsequent surveillance within 5 years after baseline colonoscopy among Medicare beneficiaries with a colonoscopy with polypectomy between 1998 and 2003.

Risk Factor		Estimate (CI)
Gender		
	Female	1 (referent)
	Male	0.83 (0.77–0.90)¥
Age group		
	66–69 years	1 (referent)
	70–74 years	1.13 (0.99–1.29)
	75–79 years	1.48 (1.29–1.69)¥
	80–84 years	1.80 (1.55–2.09)¥
	85 years and older	3.65 (3.01–4.43)¥
Race		
	White	1 (referent)
	Black	0.89 (0.75–1.06)
	Other	0.99 (0.87–1.11)
Charlson comorbidity		
	0	1 (referent)
	1	1.37 (1.24–1.52)¥
	2+	2.16 (1.97–2.36)¥
Cohort		
	1998–1999	1 (referent)
	2000–2001	1.34 (1.22–1.47)¥
	2002–2003	1.82 (1.66–1.99)¥
Urban/rural status		
	Urban	1 (referent)
	Rural	0.95 (0.83–1.08)
	Missing	0.55 (0.28–1.08)

¥ Statistically significant (p<0.01).

**Figure 2:**
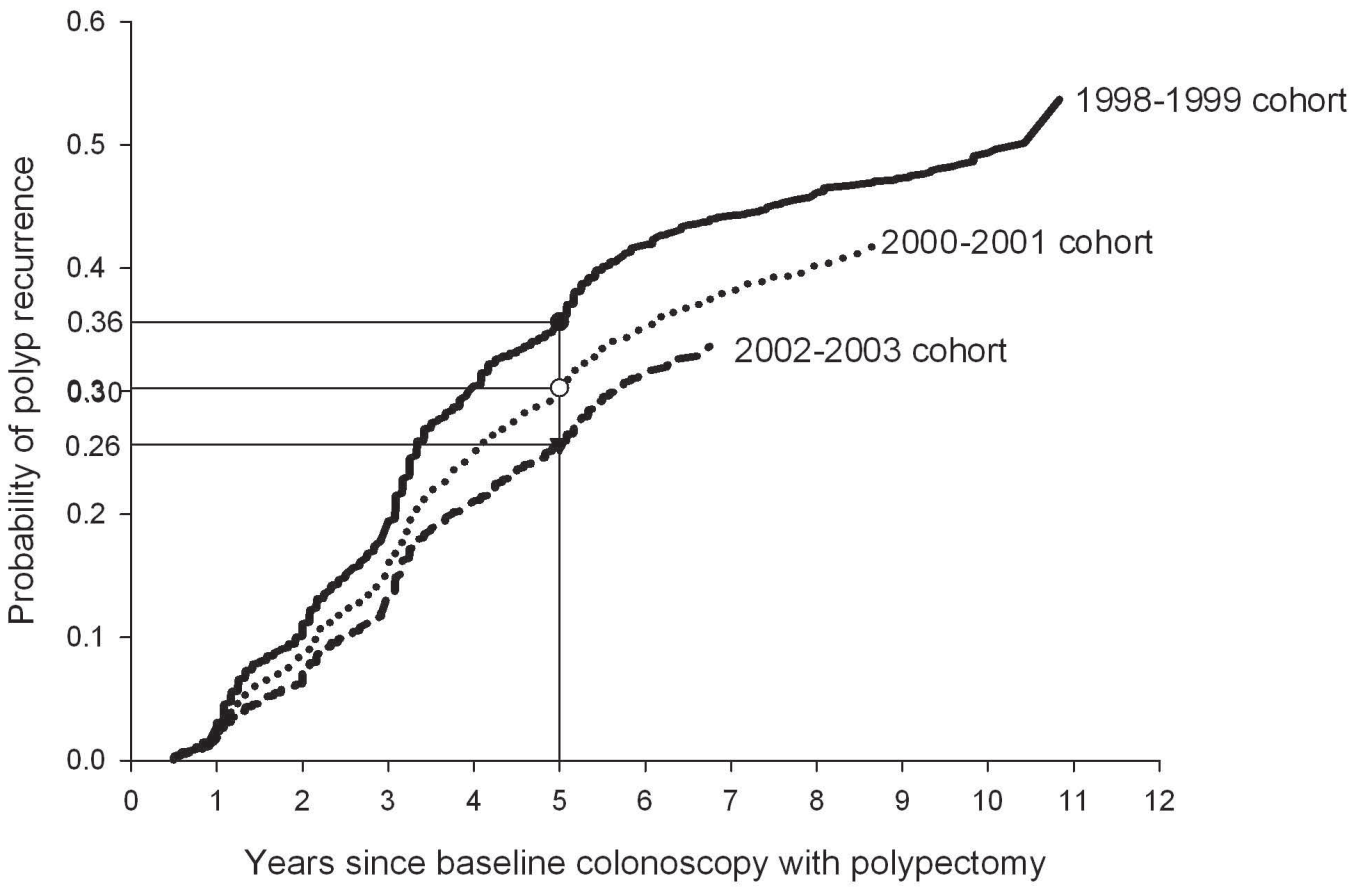
Kaplan-Meier estimates of polyp recurrence as indicated by surveillance polypectomy, stratified by cohort based on date of baseline colonoscopy with polypectomy.

The cumulative probability of polyp recurrence (Figure 2) within 5 years also significantly decreased from 36% in the 1998–1999 cohort to 26% in the 2002–2003 cohort (p<0.001). However, these estimates are still high, showing that 58% of all surveillance events within 5 years result in another polypectomy. Among people with a surveillance event within 5 years, male gender and early cohort were both associated with higher polyp recurrence rates (Table 3), with male gender being the most important one. The odds ratio for polyp recurrence in men compared to women was 1.4 (95% CI: 1.2–1.5). Age, race, rural status and comorbidity status were not significantly associated with polyp recurrence.

**Table 3:** Odds ratio for polyp recurrence as indicated by surveillance polypectomy among Medicare beneficiaries with a baseline colonoscopy with polypectomy between 1998 and 2003 and a surveillance event within 5 years of that baseline colonoscopy.

Risk Factor		Estimate (CI)
Gender		
	Female	1 (referent)
	Male	1.38 (1.24–1.54)¥
Age group		
	66–69 years	1 (referent)
	70–74 years	0.97 (0.82–1.15)
	75–79 years	0.99 (0.83–1.17)
	80–84 years	0.85 (0.69–1.05)
	85 years and older	0.89 (0.65–1.20)
Race		
	White	1 (referent)
	Black	0.77 (0.60–0.98)*
	Other race	1.10 (0.93–1.31)
Charlson comorbidity		
	0	1 (referent)
	1	1.09 (0.95–1.25)
	2+	1.33 (1.17–1.51)¥
Cohort		
	1998–1999	1 (referent)
	2000–2001	0.92 (0.82–1.05)
	2002–2003	0.88 (0.77–1.00)
Urban/rural status		
	Urban	1 (referent)
	Rural	1.07 (0.89–1.30)
	Missing	1.56 (0.63–3.88)

¥ Statistically significant (p<0.01).

* Statistically significant (p<0.05).

### Sensitivity Analysis

Our results were robust for the sensitivity analyses we performed. The 5-year probabilities of surveillance and polyp recurrence stayed within 6%-points of the original estimates (Table 4). Results were most influenced by limiting the inclusion of individuals to their first polypectomy (probability of surveillance event within 5 years decreased from 45% to 40% for the 2002–2003 cohort), and inclusion of cancer cases (probability of surveillance event increased from 58% to 64% for the 1998–1999 cohort). In all sensitivity analyses, the risk factors for failure to undergo surveillance and polyp recurrence from the GEE models did not change (data not shown).

**Table 4:** Probability of first surveillance event and first polypectomy event within 5 years after baseline colonoscopy with polypectomy among Medicare beneficiaries, stratified by cohort based on date of baseline colonoscopy with polypectomy – results of sensitivity analyses (estimated using the Kaplan-Meier method).

Analysis*	1998–1999 cohort	2000–2001 cohort	2002–2003 cohort
*Surveillance event*			
Base case^1^	58%	52%	45%
Include people with baseline sigmo^2^	60%	54%	46%
Single inclusion of individuals^3^	58%	49%	40%
Only colonoscopy surveillance^4^	57%	51%	44%
Include cancer cases^5^	64%	55%	45%
*Polyp recurrence*			
Base case^1^	36%	30%	26%
Include people with baseline sigmo^2^	36%	31%	26%
Single inclusion of individuals^3^	36%	28%	22%
Only colonoscopy surveillance^4^	36%	30%	26%
Include cancer cases^5^	42%	33%	26%

*Results in the table refer to the following analyses: 1) original analysis; 2) Inclusion of patients with polyps detected and removed at (procto-) sigmoidoscopy; 3) Single inclusion of individuals in the cohort of their first colonoscopy with polypectomy between 1998 and 2003; 4) Limiting the definition of a surveillance event to a colonoscopy; 5) Including people from the SEER-Medicare data with a cancer diagnosis.

## Discussion

This study shows that overuse of surveillance within 3 years after a polypectomy decreased from 31.5% for Medicare beneficiaries with a baseline polypectomy in 1998–1999 to 20% in 2002–2003. However, at the same time underuse of surveillance increased from 42% of Medicare beneficiaries with polypectomy in 1998–1999 not receiving surveillance within 5 years to 55% 2002–2003. Especially, women, the elderly and people with serious comorbidities were less likely to receive timely surveillance. This study also shows that timely surveillance is important, because approximately 60% of Medicare beneficiaries with polypectomy and subsequent surveillance are found to have another polyp. Polyp recurrence is especially high in the male Medicare population.

Our results were robust to alternative inclusion criteria explored in the sensitivity analyses. Limiting inclusion of individuals to their first colonoscopy with polypectomy between 1998 and 2003 decreased the probability of surveillance the most (by 5%-points in the 2002–2003 cohort). This was expected, because in this sensitivity analysis, patients that undergo regular surveillance were excluded from later cohorts, leaving patients with irregular surveillance overrepresented. Including cancer cases and assuming that all cancer diagnoses between 6 months and 5 years after polypectomy were surveillance detected cancers, increased probability of surveillance the most, by 6%-points. It is unlikely that all cancer cases were surveillance detected, but even under this extreme assumption, the probability of surveillance did not exceed 64% and was still decreasing over time to 45% in the 2002–2003 cohort.

With the introduction of Medicare coverage of colonoscopy screening for high-risk individuals in 1998, we anticipated an increase in surveillance rates for this population but found decreased rates. Possible explanations for this unexpected finding include the growing recognition that surveillance in many settings can and should be done less frequently (e.g. every 10 years in case of 1 tubular adenoma) [13]. Furthermore, reimbursement of colonoscopy screening may have triggered a different selection of people to undergo colonoscopy. In the early years, our study probably consisted mostly of high-risk patients with a family history of CRC, while in later years more people without family history (and lower likelihood of advanced adenomas) were likely included in our study as Medicare expanded colonoscopy coverage from only high-risk individuals in 1998 to all individuals in 2001. Patients with family history are more likely to have advanced adenomas and these two factors have been shown to synergistically influence colonoscopy utilization [6]. The later cohort may not be deemed as high-risk by treating physicians, compared with the original set of people with symptoms and family history, and may therefore not receive an intensive surveillance recommendation. Another explanation for the decrease in surveillance rates might be the improvement in the quality of colonoscopy, such as use of high-definition endoscopes and use of split-dose preparation. As a consequence of these techniques, treating physicians may be more confident of having completely cleared the colon, recommending longer surveillance intervals. In addition, higher quality colonoscopy may have resulted in increased detection rates of diminutive polyps. Diminutive polyps are more often non-neoplastic [14] and people with non-neoplastic polyps are not recommended to undergo regular surveillance [2]. Both explanations are supported by the increasing number of people receiving baseline colonoscopy with polypectomy (33% increase from 1998–1999 to 2002–2003) likely influencing the composition of the population. However, both also remain speculative and need further investigation.

Our estimates for the probability of inappropriate surveillance within 3 years (20–31%) are considerably lower than those reported in surveys among primary care physicians and endoscopists: in these surveys more than 50% of physicians recommended surveillance within 3 years for people with a hyperplastic polyp or small tubular adenomas only [15], [16]. More recent estimates based on medical chart review indicate that approximately 24% of patients with hyperplastic polyps only were recommended to undergo surveillance within 4–6 years and 35% of patients with only small adenomas were recommended to return within 1–3 years [17], which is more in line with our estimates.

Our estimates for the probability of a surveillance event within 5 years for Medicare beneficiaries in the 2002–2003 cohort (45%) are considerably lower than those found by Amonkar et al (74%) [8] in a study cohort with index colonoscopies in 1994. Even though our findings differ from those of Amonkar et al., our results mirror those of Cooper at al. [18], whose study cohort reflects the same timeframe. Cooper et al. found a decreasing trend in surveillance utilization for more recent index colonoscopies. Thus, practice pattern changes in more recent years are a very plausible explanation. Possible explanations for the difference between our results and those of Amonkar et al. are the inclusion of people with prevalent polyps (i.e. a diagnosis of colorectal polyp before the baseline colonoscopy) in Amonkar's study and the exclusion of individuals with gastrointestinal comorbidities for which colonoscopy may constitute part of standard work up in our study. Our findings are also consistent with findings from studies of surveillance of adenoma patients in other health care settings. Laiyemo et al. found a probability of a surveillance colonoscopy in adenoma patients of 59.7% after a mean follow-up time of 5.9 years [5]. In the study by Schoen, surveillance probabilities after 5 years varied from 46.7% to 58.5% depending on the number and type of adenomas found [6].

Risk factors for failure to receive subsequent surveillance are also the ones associated with the lowest polyp recurrence rates: later cohort and women. The lower polyp recurrence in later cohorts can be explained by higher failure rates to undergo a surveillance event. Interestingly age was not found to be a predictor for polyp recurrence, while older age has been consistently found to be an independent predictor for (advanced) adenoma recurrence [19], [20]. When age is investigated as a potential risk factor, age is often dichotomized into younger than 60 and 60 years and older [20]. The fact that all individuals in our study were into the latter category, might explain why we did not see an age effect. Amonkar et al found polyp recurrence to decrease with the age of the patient in Medicare [8]. They suggested that frailty of the patient might play a role, and that physicians suggest less aggressive treatments for patients in the oldest age groups, because of increased risk of complications [21]. It has been suggested that removal of diminutive polyps could be foregone, especially in older individuals as death from other causes is likely to occur before these polyps become invasive tumors [22].

Several limitations are noteworthy. First, this study is based on administrative data that were not collected for research purposes. As a consequence, the data may contain errors due to billing and coding. However, a study investigating the accuracy of Medicare claims for measuring colorectal endoscopy use concluded that Medicare claims can provide accurate information on whether a patient has undergone colorectal endoscopy and may be more complete than physician medical records [9]. A second study investigating the accuracy of Medicare claims for identifying findings and procedures performed during colonoscopy concluded that Medicare claims have high sensitivity and specificity for polyp detection, biopsy, and polypectomy at colonoscopy [23].

Second, it cannot be distinguished from Medicare data whether subsequent colorectal examinations were performed for surveillance purposes or for clinical reasons. This was also confirmed by Schenck et al., suggesting that researchers who use Medicare claims to assess rates of colorectal testing should include both screening and diagnostic endoscopy procedures in their analyses [9]. In this analysis, we included both types of procedures as recommended, but we tried to exclude colorectal examinations for clinical reasons by excluding people with comorbidities prior to baseline colonoscopy that may require repeat colonoscopies for reasons other than adenoma findings. As a result of that exclusion, the population in this study will be somewhat healthier than the average Medicare population. Third, our study only includes patients enrolled in fee-for-service and not HMO. HMO patients are shown to have higher stage at diagnosis for CRC, which may indicate lower utilization since 2000 [24].

Fourth and most importantly, Medicare claims data lack information on clinical polyp characteristics such as number of polyps and size and histology of the polyps removed. As a result not all beneficiaries in our cohorts may have actually had an adenoma removed, but could also have had a non-adenomatous polyp removed. Surveillance exams are not recommended for people with these types of polyps [2], and we may have therefore underestimated the probability of timely surveillance in our analysis. In several studies in an average-risk (screening) population, approximately half of people with polyps have been found to have non-adenomatous polyps only [14], [25], [26]. In our study approximately 50% of patients with polypectomy did not receive surveillance. In theory, these could all be individuals with non-adenomatous polyps only, and then the probability of timely surveillance would actually be near-perfect. Given the careful sensitivity analyses performed and the consistencies between our estimated surveillance probabilities and that of other community-based studies [5], [6], near-perfect surveillance is unlikely.

In conclusion, our study shows that expansion of Medicare reimbursement for colonoscopy screening in high-risk individuals in 1998 has not reduced the underutilization of surveillance in the Medicare population. Surveillance rates after polypectomy have further declined between 1998 and 2003. Measures should be taken to increase surveillance uptake, because polyp recurrence is substantial in this population.

## Supporting Information

Appendix S1 **Overview of Medicare beneficiaries included in the study and how they are followed over time based on six hypothetical examples.**
(TIF)
